# The Effect of Interferon Type I Adjuvant Therapy on the Lifespan and Complications of Glioma Patients Undergoing Chemotherapy: A Systematic Review and Meta‐Analysis

**DOI:** 10.1002/cnr2.70507

**Published:** 2026-03-12

**Authors:** Nima Goudarzi, Abolfazl Sam Daliri, Arshia Harati, Shabnam Soheil Nader, Kourosh Kabir

**Affiliations:** ^1^ School of Medicine, Alborz University of Medical Sciences Karaj Iran; ^2^ Department of Neurology Alborz University of Medical Sciences Karaj Iran; ^3^ Department of Community Medicine Iran University of Medical Sciences Tehran Iran

**Keywords:** adverse events, alkylating drugs, glioma, interferon type I, interferon‐alpha, interferon‐beta, meta‐analysis, overall survival, progression‐free survival, temozolomide

## Abstract

**Background:**

The malignant glioma, as the most common and aggressive primary brain and spinal cord neoplasm, has shown limited responsiveness to available treatments, including tumor dissection, radiation, and chemotherapy. Thus, interferon type I, as a supplemental therapy, is added to the main therapies to overcome neoplasm resistance and prolong the patients' lifespan.

**Methods:**

To clarify the effects of interferon adjuvant therapy on the lifespan and complications of glioma patients, we conducted a systematic review and meta‐analysis by searching valid databases, scanning, and screening full texts based on a predefined protocol for the study.

**Results:**

Seven studies were eligible for data synthesis and analysis. Lifespan, tumor progression, adverse events, and genetic factors were studied and analyzed in this systematic review and meta‐analysis. The median overall survival (OS) and median progression‐free survival (PFS) were increased as a result of the supplemental therapy. However, only the median OS was significantly improved (OS: HR = 0.74, 95% CI [0.58, 0.96]; *p* = 0.02/PFS: HR = 0.93, 95% CI [0.74, 1.18]; *p* = 0.56). Additionally, interferon adjuvant therapy could affect the toxic events of alkylating drugs; Flu‐like and neurological events were significantly exacerbated (odds ratio = 3.31, 95% CI [1.20, 9.08]; *p* = 0.02, odds ratio = 6.15, 95% CI [2.20, 17.22]; *p* = 0.0005), while dermatological events were effectively alleviated as a result of interferon therapy (odds ratio = 0.29, 95% CI [0.10, 0.84]; *p* = 0.02). Variation of the hematological and hepatic events was not statistically significant (odds ratio = 1.06, 95% CI [0.52, 2.17]; *p* = 0.87, odds ratio = 1.06, 95% CI [0.67, 1.66]; *p* = 0.81).

**Conclusion:**

Despite the development of a few adverse events, interferon type I supplemental therapy in combination with radiation and chemotherapy could significantly extend the lifespan of glioma patients.

**Abbreviations**
ACNU: 3‐[(4‐amino‐2‐methyl‐5‐pyrimidinyl) methyl‐l‐(2‐chloro‐ethyl)‐l‐nitrosourea hydrochloride
BCNU: Bis‐chloroethyl nitrosourea, Carmustine
CI: confidence interval
GBM: glioblastoma
HGG: high‐grade glioma
HR: hazard ratio
IFN: interferon
MCNU: 1‐(2‐chloroethyl)‐1‐nitroso‐3‐([(2R,3S,4S,5R,6S)‐3,4,5‐trihydroxy‐6‐methoxyoxan‐2‐yl]methyl)urea, Ranimustine
MGMT: O(6)‐methylguanine‐DNA methyltransferase
OS: overall survival
PFS: progression‐free survival
RCT: randomized controlled trial
RevMan: review manager
RT: radiotherapy
TMZ: temozolomide
TTP: time to tumor progression

## 1 Introduction

Glioma grades 3 and 4, defined as high‐grade glioma by the World Health Organization, contain a vast spectrum of malignant brain tumors (including gliosarcoma, glioblastoma, anaplastic glioma, anaplastic oligodendroglioma, and anaplastic oligoastrocytoma) that are the most common primary central nervous system neoplasms (approximately 26%) [1, 2]. Investigations have demonstrated that the annual age‐standardized incidence of glioma is approximately 3 cases per 100 000 people. Additionally, men have more exposure to this neoplasm than women [3]. The incidence of glioma varies among different populations worldwide, and it has been concluded that genetic predisposition and heredity are significant factors in the development of the disease [3]. Certain genetic conditions, like Li‐Fraumeni syndrome, neurofibromatosis, and Turcot syndrome, can increase the risk of glioblastoma (GBM) [4, 5, 6]. Additionally, factors like aging, gender (male), and exposure to radiation and chemicals such as petroleum, pesticides, synthetic rubber, and vinyl chloride can increase the risk of glioma [7, 8, 9]. The survival rate and prognosis of the disease depend on the molecular and genetic subtypes of the tumors. There are four molecular tumor subtypes: classical, mesenchymal, proneural, and neural, with the proneural subtype being associated with a better prognosis. Genetic studies have shown that mutations in IDH1, IDH2, and TERT, along with MGMT methylation, are associated with a more favorable prognosis [10, 11]. In contrast, gain of chromosome 7 and loss of chromosome 10 are associated with poorer prognosis [12]. Among all types of malignant glioma, glioblastoma accounts for 70%–75% of diffuse gliomas, and the median overall survival (OS) of patients ranges from 14 to 17 months; therefore, the duration between diagnosis and mortality is very short [13]. Furthermore, glioma could establish many life‐threatening events, including brain hemorrhage, brain herniation, hydrocephalus, and seizures [14, 15, 16].

High mortality rate of malignant gliomas has resulted in extensive efforts to increase the patient's lifespan. Tumor dissection, radiation, and chemotherapy are the primary treatment methods used to prolong the lifespan of patients [17, 18]. Chemotherapy has had impressive development as a treatment method for neoplasms. In this way, alkylating drugs containing Carmustine (BCNU), ACNU, and Ranimustine (MCNU) have been used until the 2000s. Currently, temozolomide (TMZ) is the most common chemotherapeutic drug [19, 20, 21, 22].

High‐grade glioma (HGG), similar to other neoplasms, could be resistant to therapeutic methods such as radiation and chemotherapy, so novel therapies are needed to overcome treatment resistance in glioma [23]. Nevertheless, many methods, including vaccination, interferon (IFN), ribavirin, and other supplemental therapies, have been discovered that may overcome the resistance of gliomas [24, 25]. Based on randomized controlled trials (RCTs), IFN is one of the supplements used in combination with alkylating drugs that may help increase the patient's lifespan and prevent tumor progression [19, 26, 27, 28]. However, this novel supplemental therapy could affect the rate of chemotherapeutic side effects, including hepatic, hematologic, neurological, and flu‐like symptoms [29, 30, 31].

Given the conflicting findings from previous studies, the efficacy and safety of IFN adjuvant therapy in lifespan prolongation remain unclear. Additionally, different subtypes of IFN are associated with varying adverse events in the patient's treatment. Therefore, this systematic review and meta‐analysis aim to provide clearer insights about the potential benefits and risks of IFN‐based adjunctive therapy combined with alkylating drugs in glioma patients. It will also clarify the characteristics of IFN subtypes.

## 2 Materials and Methods

### 2.1 Search Strategy

In this systematic review and meta‐analysis, RCT studies were investigated and analyzed. The study was conducted per PRISMA statement guidelines. Ethical approval was not necessary for this manuscript due to its review format. Two reviewers (ASD and NG) independently conducted the database search (PubMed, Scopus, Web of Science, and the Central) on February 18, 2024. We utilized MeSH keywords and synonyms obtained from MeSH PubMed to guide our search. The search strategy was as follows: (“Glioma”[MeSH] OR synonyms [All Fields]) AND ((“Antineoplastic Agents, Alkylating” [Pharmacological Action] OR synonyms [All Fields]) AND (“Interferons”[MeSH] OR synonyms [All Fields])).

### 2.2 Study Selection Criteria

The inclusion criteria were selected based on the protocol established using the PICOT framework. The studies included only RCTs that investigated newly diagnosed glioma patients (astrocytomas, supratentorial GBM, gliosarcoma, anaplastic gliomas, anaplastic oligoastrocytomas, and anaplastic oligodendroglioma according to World Health Organization (WHO) criteria). The intervention group of the studies received intravenously or subcutaneously interferon type I + Alkylating drugs (TMZ, ACNU, BCNU, or MCNU) + Radiation. In contrast, the comparison group received only Alkylating drugs. The outcomes measured in the studies included lifespan criteria (OS, progression‐free survival (PFS), time to tumor progression (TTP), survival rate) and adverse events (hematological, flu‐like, dermatological, neurological, hepatic events).

The exclusion criteria included other types of brain tumors (medulloblastoma, meningioma, ependymoma, and metastatic tumors) and different kinds of interferons. Furthermore, animal and culture studies were excluded. All other publication types, such as reviews, book chapters, books, conference papers, short surveys, notes, editorials, retracted papers, and letters, were excluded from the analysis.

**FIGURE 1:**
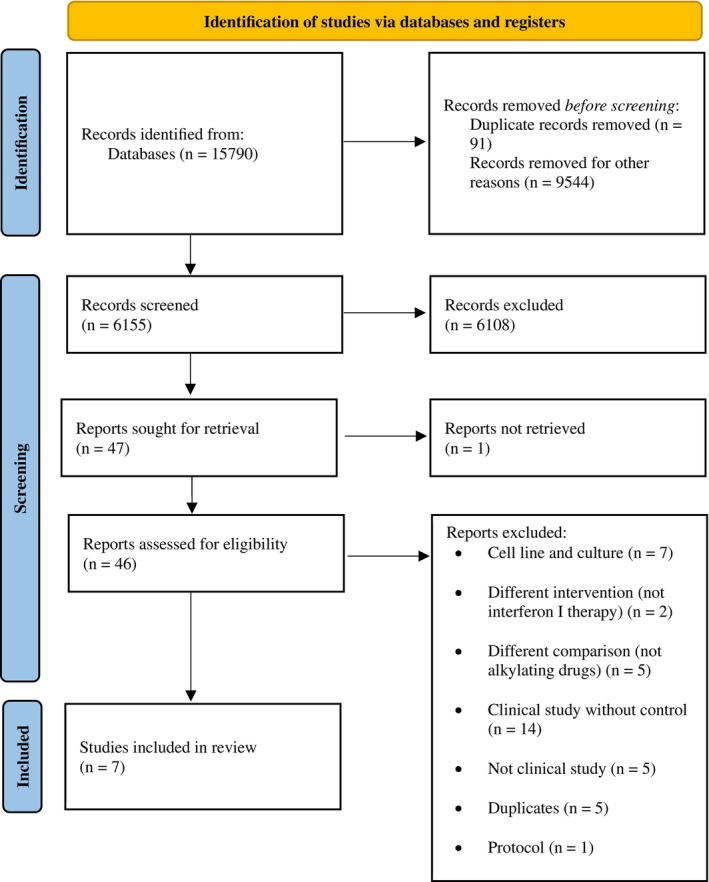
Flow chart of study selection.

The title and/or abstract of the remaining records were scanned independently by two authors (A.S.D., N.G.); the differences between the two arms of scanning were resolved by a third investigator (A.H.). Ultimately, 46 articles were selected for full‐text screening. Of these, seven records were suitable for data synthesis and analysis (Figure 1).

### 2.3 Data Extraction

Two authors (A.S.D., N.G.) conducted the data extraction process by reading and thoroughly investigating the full texts. The extracted data is classified based on PICOT, which includes age, gender, sample size, type of intervention, and comparison (type and dosage), and study outcomes containing OS, PFS, adverse events, TTP, survival rate, and MGMT subgroups.

### 2.4 Quality Assessment

ASD and NG independently evaluated the risk of bias according to the Cochrane risk of bias tool 2 (RoB2/Review Manager software, Version 5.4.1, The Cochrane Collaboration, 2020). Any discrepancies were resolved by a third investigator (A.H.). According to *review manager* (*RevMan*), selection bias (random sequence generation and allocation concealment), performance bias (blinding of participants and personnel), detection bias (blinding of outcome assessment), attrition bias (incomplete outcome data), reporting bias (selective reporting), and other bias (follow‐up patients) were considered for quality assessment of studies. The bias was reported in three levels, including low risk (green), non‐obvious risk (yellow), and high risk (red).

### 2.5 Data Synthesis and Analysis

The outcomes of selected studies were analyzed by the Review Manager assistant tool (Review Manager software, Version 5.4.1, The Cochrane Collaboration, 2020). The analyzed outcomes included OS, PFS, and adverse events. OS and PFS, reported using the Cox hazard model and multivariable analysis, were analyzed using a generic inverse variance statistical model and a fixed‐effect hazard ratio with a 95% confidence interval. The adverse events, reported with percentages and numbers of incidences, were analyzed using a dichotomous Mantel–Haenszel statistical model and a random‐effects odds ratio with a 95% confidence interval. The adverse events were classified as hematological (anemia, lymphopenia, neutropenia, leukopenia, and thrombocytopenia), flu‐like (fever, nausea, emesis, fatigue, myalgia, chill, pulmonary, and headache), neurological (epileptic), hepatic, and dermal events. The mean incidence of the hematological and flu‐like events (due to having subgroups) was calculated and estimated by taking the average and rounding decimals up or down (X ≥ 0.5 rounds up, X < 0.5 rounds down).

### 2.6 Quality of Evidence

This systematic review and meta‐analysis investigated the effect of interferon adjuvant therapy on OS, PFS, and complications of newly diagnosed glioma patients. Given the limited number of studies, the analysis of OS, PFS, and dermatological side effects had moderate certainty of evidence. The remaining analysis had a high level of certainty due to complete data reporting, proper randomization, and an appropriate number of studies and participants (Table 1).

**TABLE 1:** Quality of evidence.

Outcome		Predicted absolute effects		HR (first two) odds ratio (the last) with 95% CI	Number of participants	GRADE; certainty of the evidence
		Outcomes in intervention	Outcomes in control			
OS		—	—	0.74 [0.58, 0.96]	320 (2 RCTs)	⨁⨁⨁◯ ^a^
PFS		—	—	0.93 [0.74, 1.18]	320 (2 RCTs)	⨁⨁⨁◯ ^a^
Averse events	Hematological	61 per 100	60 per 100	1.06 [0.52, 2.17]	647 (4 RCTs)	⨁⨁⨁⨁
	Flu‐like	28 per 100	9 per 100	3.31 [1.20, 9.08]	647 (4 RCTs)	⨁⨁⨁⨁
	Dermatological	3 per 100	10 per 100	0.29 [0.10, 0.84]	320 (2 RCTs)	⨁⨁⨁◯ ^a^
	Neurological	10 per 100	2 per 100	6.15 [2.20, 17.22]	474 (4 RCTs)	⨁⨁⨁⨁
	Hepatic	21 per 100	20 per 100	1.06 [0.67, 1.66]	647 (4 RCTs)	⨁⨁⨁⨁

*Note:* Studies that investigated the efficacy of interferon adjuvant therapy on OS, PFS, and adverse events. Population: Glioma patients. Intervention: IFN type I + alkylating drugs. Comparison: Alkylating drugs. GRADE: Working Group Grades of Evidence. HIGH: There is complete confidence that the true effect is close to the estimate of the effect. ⨁⨁⨁⨁. MODERATE: The effect of the estimate is moderately confided: Despite of the true effect is likely close to the effect estimate, it could be substantially different from the effect estimate. ⨁⨁⨁◯. LOW: The validity of the effect estimate is low: The true effect may be substantially different from the estimate of the effect. ⨁⨁◯◯. VERY LOW: Very little confidence exists in the effect estimate: The true effect is probably different from the effect estimate. ⨁◯◯◯. Low participant (one downgrade for imprecision) [32]. Studies report data incompletely or incorrectly (one downgrade for risk of bias) [33]. Proper randomization (one downgrade for risk of bias) [34].

^a^ The number of RCT studies used in meta‐analysis is less than 3 numbers (one downgrade for comprehensiveness).

## 3 Results

### 3.1 Study Selection

A total of 15 790 records were obtained by searching the mentioned databases (PubMed, Scopus, Web of Science, and Central) with keywords (MeSH and synonyms) based on the study protocol (PICOT). Seven thousand seven hundred and eighty‐two reviews, 1104 book chapters, 371 books, 111 conference papers, 103 short surveys, 91 duplicates, 28 notes, 25 editorials, 13 retracted papers, and seven letters were removed before screening. Thus, 6155 records remained and were scanned by screening the title and/or abstract. Furthermore, one manuscript (Nagai 1991) could not be retrieved because it was published in a domestic Japanese journal, and even the Japanese full‐text was unavailable in the databases; also, the full‐text could not be obtained by communication with the author [35]. Forty‐six records were selected for investigating the full‐texts based on the eligibility criteria and the protocol. Ultimately, 39 records were excluded for the reasons mentioned in Figure 1, and 7 studies (Guo 2023, Natsume 2020, Wakabayashi 2018, Buckner 2001, Taylor 1998, Harada 1996, Yoshida 1994) were selected for data synthesis and analysis. Natsume (2020) and Wakabayashi (2018), as well as Buckner (2001) and Taylor (1998), reported different results from a standard sample; thus, these studies were considered a common study for data synthesis and analysis. Therefore, all the selected studies were used for data synthesis, from which Guo (2023), Natsume (2020), Wakabayashi (2018), Buckner (2001), Taylor (1998), and Harada (1996) were analyzed based on available outcomes.

### 3.2 Study Characteristics

All seven selected studies were RCTs from which 2 [26, 36] and 3 [19, 27, 28]. The studies were phase II and phase III clinical trials, respectively. The study phases of Harada (1996) and Yoshida (1994) were non‐obvious [18, 37] (Table 2).

**TABLE 2:** Study characteristics.

Author, year	Country	Study design	Sample size (intervention/control)	Gender (male/female)	Age (intervention/control)	Type of intervention			Type of comparison (dosage and cycle)
						Interferon (dosage and cycle)	Alkylating drug (dosage and cycle)	Radiation (total dosage)	
Guo 2023 [19]	China	RCT	100/99	120/79	46/46.8	IFN‐α (3 million U on days 1, 3, and 5 every 28 days)	TMZ (150–200 mg/m^2^ on days 2–6 every 28 days)	60 Gy	TMZ (150–200 mg/m^2^ on days 1–5, every 28 days for a maximum of 12 cycles)
Wakabay ashi 2018 and Natsume 2020 [26, 36]	Japan	RCT	59/63	73/49	61/61	Intravenously IFN‐β (3 MU/body on day 1, day 3, and day 5 every 28 days)	TMZ (100–200 mg/m^2^/day on days 1–5 every 28 days)	60 Gy	TMZ (75 mg/m^2^, daily) followed by maintenance of TMZ (100–200 mg/m^2^/day, days 1–5, every 4 weeks)
Buckner 2001 and Taylor 1998 [27, 28]	USA	RCT	138/137	214/146^b^	57^a^	Subcutaneously IFN‐α (12 million U/m^2^ on Days 1–3 of Weeks 1, 3, and 5 of each 7‐week cycle)	Intravenously BCNU (150 mg/m^2^, every 7 weeks BCNU was given on Day 3 after the IFN‐a injection)	64.8 Gy	Intravenously BCNU (200 mg/m^2^, on Day 1 every 7 weeks for up to 1 year to a maximum of 6 cycles)
Harada 1996 [37]	Japan	RCT	24/28	26/26	46.1/51.6	IFN‐β (2 million IU/m^2^, 5 times weekly for 8 consecutive weeks)	Intravenous MCNU (2 mg/kg, at the time of the radiation)	50–60 Gy	Intravenous MCNU (2 mg/kg at the time of the radiation)
Yoshida 1994 [18]	Japan	RCT	60/33	—	—	Intravenously IFN‐β (1–3 million U once a day for 7 days)	Intravenously ACNU (3 mg/kg twice at six weekly intervals)	40–60 Gy	Intravenously ACNU (3 mg/kg twice at six weekly intervals)

^a^ The separate mean age of the intervention and control groups was not available.

^b^ Before randomization.

Participants included newly diagnosed glioma patients (astrocytomas, supratentorial GBM, gliosarcoma, anaplastic gliomas, anaplastic oligoastrocytomas, and anaplastic oligodendroglioma) according to World Health Organization criteria, who had not received prior chemotherapy, radiotherapy, or immunotherapy for their brain tumor. A total of 740 patients (532 men and 208 women) participated in the trials, with 380 and 360 patients assigned to the intervention and control groups, respectively. The domain age of onset varied from 46 to 61 years old (Table 3).

**TABLE 3:** Study outcomes and tumor details.

Author, year	Outcomes (intervention/control)			Tumor details		
	OS	PFS	Adverse events	Tumor type	Chemotherapy	Performance
Guo 2023 [19]	26.7 [21.6–31.7]/18.8 [16.9–20.7] (months)	14.8 [12.3–17.4]/12.9 [11.8–14.0] (months)	Hematological/Flu like/Epilepsy/Dermal toxicity/Hepatic	Newly diagnosed HGG (WHO grades 3 and 4 astrocytomas, including supratentorial GBM, gliosarcoma, anaplastic gliomas, anaplastic oligoastrocytomas, and anaplastic)	No prior chemotherapy, radiotherapy, immunotherapy	WHO Karnofsky performance status of at least 60%
Wakabay ashi 2018 and Natsume 2020 [26, 36]	24.0 [18.8–27.4]/20.3 [15.4–26.9] (months)	8.5 [6.6–11.9]/10.1 [7.5–11.8] (months)	Hematological/Flu like/Dermal toxicity/Hepatic	Newly diagnosed GBM based upon WHO 2007 (IARC 4th edition); 50% of the tumor located in supratentorial areas, without involvement of the optic, olfactory nerves, and pituitary gland and without	No history of previous chemotherapy or radiotherapy	Eastern Cooperative Oncology Group (ECOG) performance status of 0–2 or 3
Buckner 2001 and Taylor 1998 [27, 28]	300/357 (days)	^a^	Hematological/Flu like/Epilepsy/Hepatic	Supratentorial diffuse glioblastoma, gliosarcoma, anaplastic astrocytoma, or anaplastic oligoastrocytoma according to WHO criteria	No prior chemotherapy, radiation therapy, or immunotherapy	WHO grade (Grade 3 vs. Grade 4), ECOG performance status (0–1 vs. 2–3)
Harada 1996 [37]	—	6.9 ± 1.4/8.5 ± 2.0 (months)	Hematological/Flu like/Hepatic	Malignant glioma	—	Performance status (PS) of 0‐3
Yoshida 1994 [18]	—	—	—	Glioblastoma, anaplastic astrocytoma or brain‐stem glioma	—	—

^a^ TTP = 148/169 days.

Intervention and control groups, as the two arms of the study, received radiation therapy and alkylating drugs (oral or intravenous TMZ, ACNU, BCNU, and MCNU). The intervention groups received interferon type I as adjuvant therapy. Complete information, including dosage and cycle, is provided in Table 2.

### 3.3 Risk of Bias

**FIGURE 2:**
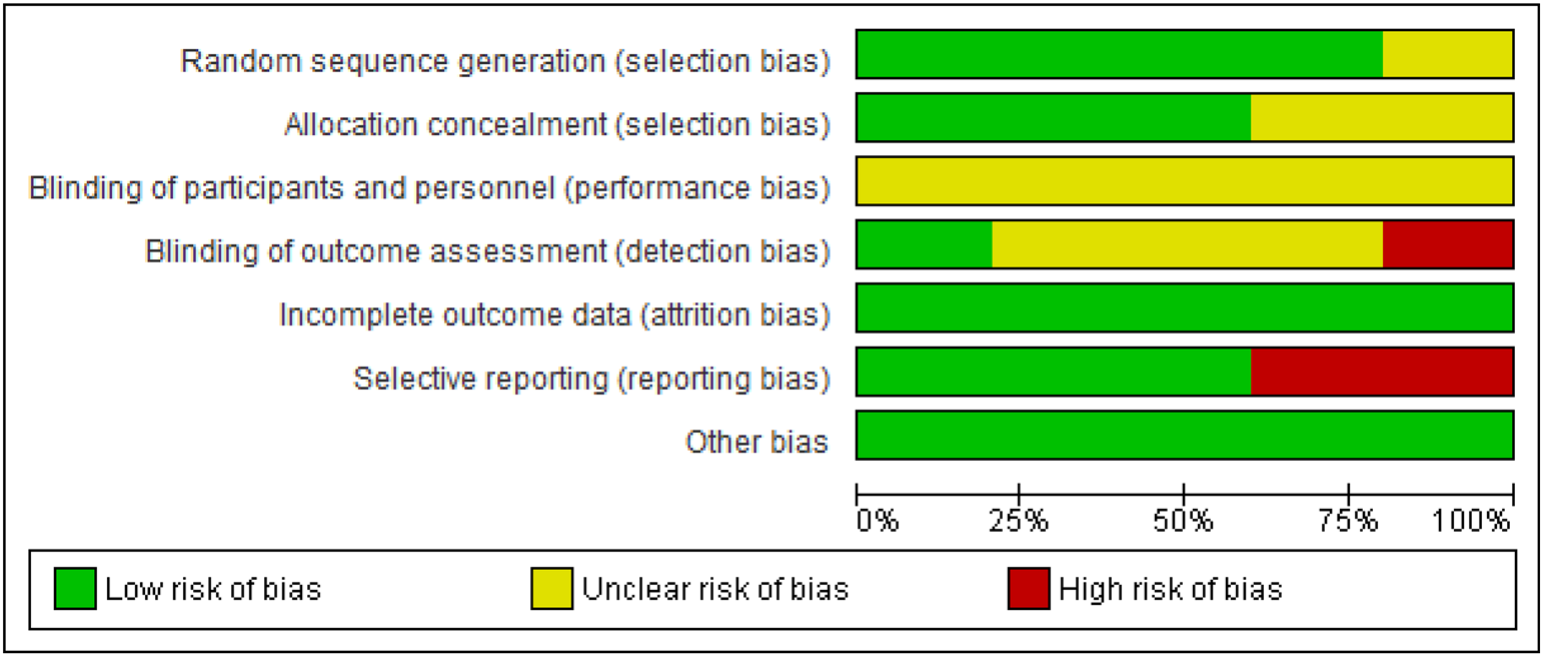
Overall risk of bias.

The pattern of risk of bias was assessed using Cochrane risk of bias tool 2 (RoB2) before investigating the articles. The random sequence generation bias was categorized as high risk if the study was neither randomized nor employed standard randomization methods (e.g., computer‐generated list, envelope, dice). Despite all the included studies being properly randomized, the Yoshida 1994 randomization method was not immediately apparent. In cases where participants were randomized into groups based on a block design, sealed envelope, and opaque, the allocation bias was considered to be of low risk. The performance and detection bias were evaluated based on whether participants, personnel, and outcome assessors were blinded, as reported in the articles. Attrition bias was measured by dropout rates exceeding 12.5% after randomization. Reporting bias was deemed high risk if there was incomplete reporting of outcomes, which was mentioned in the methodology but not fully presented in the results of the studies (Figure 2).

Harada's 1996 study was published in Japanese, so the article was translated into English using Google Translate for data extraction. This process may introduce translation errors and bias into the data collection. Additionally, some included trials had small sample sizes and variable protocols, which may limit generalizability. The risk of performance and detection bias could not be excluded in all cases.

**FIGURE 3:**
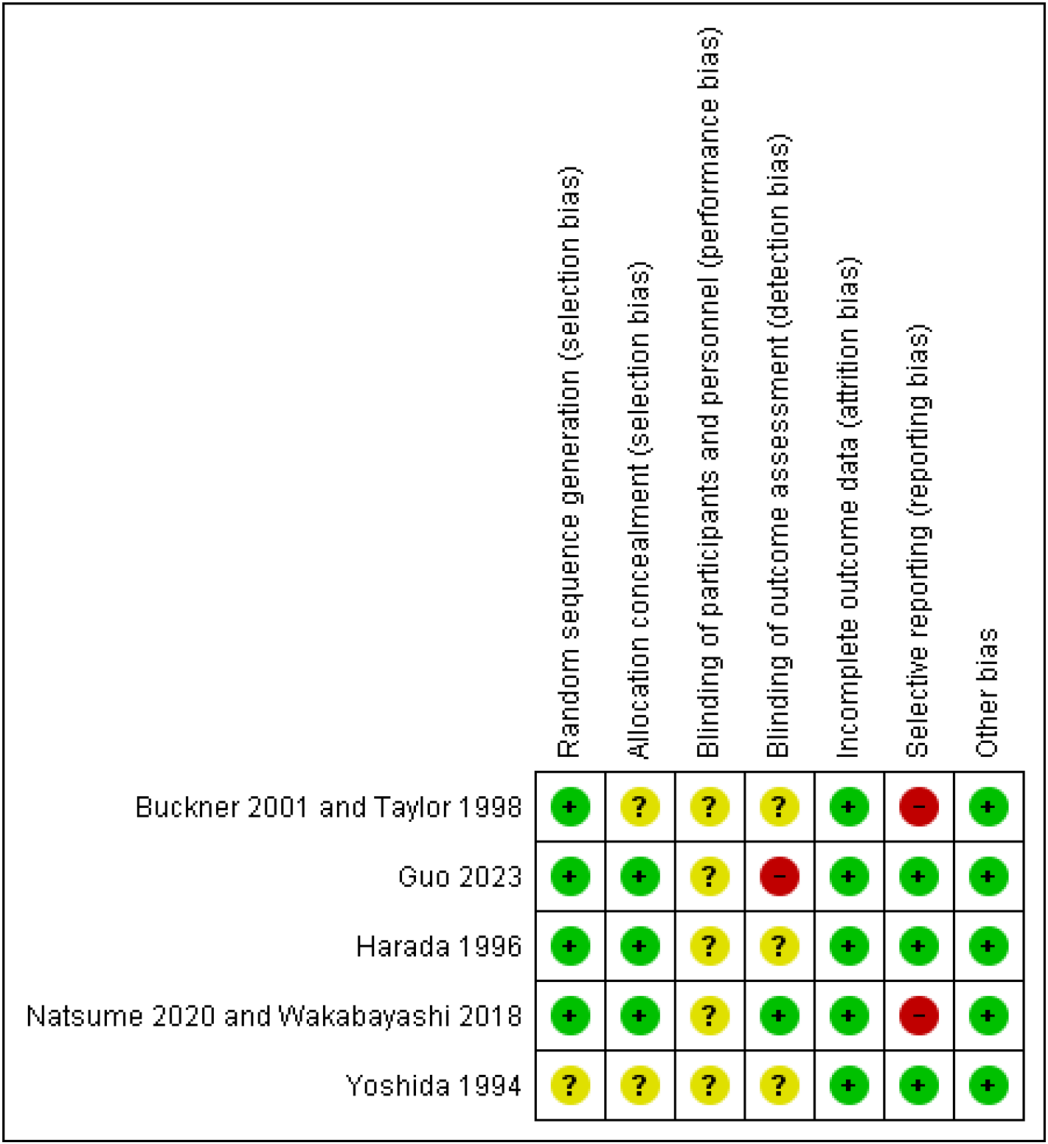
The summary of risk of the bias.

The selection bias had a low risk of bias of at least 60%, while the remaining percentage was unclear. The performance bias of all studies was ambiguous; the detection bias was high in just 20% of studies, and the residual part had a low or unclear risk of bias. The attrition and other biases had a low risk of bias, but the reporting bias was high in 40% of the studies. The details of the risk of bias are shown in Figure 3.

### 3.4 Data Synthesis

#### 3.4.1 Overall Survival (OS)

The median OS was reported as outcomes of Guo 2023, Wakabayashi 2018, Buckner 2001, and Taylor 1998 studies.

Guo et al. demonstrated that interferon‐alpha adjuvant therapy (arm I) had a positive effect in comparison to TMZ alone (common chemotherapy treatment drug, arm II); in this way, this intervention prolonged the OS in arm I compared to arm II effectively. In the WHO subgroup analysis, it was shown that this therapy had a positive effect in both grade 3 and grade 4 glioblastoma.

Wakabayashi et al. indicated the effect of interferon‐beta adjuvant therapy (arm I) compared to TMZ alone (arm II). It was shown that adjuvant therapy did not have any positive effects in prolonging the median OS of glioma patients. The subgroup analysis showed that male patients (HR 0.78), those aged ≤ 49 years (HR 0.45), and those with an ECOG PS of 0 (HR 0.74) had a better median OS in arm I compared to arm II.

Buckner and Taylor et al. demonstrated the effect of interferon‐alpha adjuvant therapy (arm I) compared to BCNU alone (arm II). It was reported that this therapy did not have a positive impact on median OS (Table 4).

**TABLE 4:** Data synthesis.

Study	Intervention (arm I)	Comparison (arm II)	Survival		
			Overall survival (months)	Survival rate	Progression free survival (months)
Guo 2023 [19]	IFN‐alpha + TMZ	TMZ alone	Median OS: Arm I: 26.7 [95% CI, 21.6–31.7] Arm II: 18.8 [95% CI, 16.9–20.7] HR 0.64 [95% CI, 0.47–0.88]; *p* = 0.005 WHO 3: HR 0.61 [95% CI, 0.37–0.99]; *p* = 0.04 WHO 4: HR 0.67 [95% CI, 0.45–0.99]; *p* = 0.04	Median 2‐year OS rates: Arm I: 57.4% [95% CI, 47.6%–67.2%] Arm II: 37.3% [95% CI, 27.7%–46.9%], The median 5‐year OS rates: Arm I: 18.1% [95% CI, 10.1%–26.1%] Arm II: 9.1% [95% CI, 2.4%–15.8%]	Arm I: 14.8 [95% CI, 12.3–17.4] Arm II: 12.9 [95% CI, 11.8–14.0] HR 0.79 [95% CI, 0.59–1.06]; *p* = 0.11 WHO 3: Arm I: 24.3 [95% CI, 21.7–27.0] Arm II: 14.1 [95% CI, 10.1–18.2] HR 0.63 [95% CI, 0.41–0.99]; *p* = 0.04 WHO 4: Arm I: 12.0 [95% CI, 9.8–14.2] Arm II: 12.8 [95% CI, 12.2–13.4] HR 1.11 [95% CI, 0.76–1.64]; *p* = 0.58
Wakabay ashi 2018 and Natsume 2020 [26, 36]	IFN‐beta + TMZ	TMZ alone	Median OS: Arm I: 24.0 [95% CI, 18.8–27.4] Arm II: 20.3 [95% CI, 15.4–26.9] HR 1.00, [95% CI, 0.65–1.55]; *p* = 0.51 Male patients: HR 0.78 [0.46–1.32] Age ≤ 49 years: HR 0.45 [0.16–1.29] ECOG PS 0: HR 0.74 [0.33–1.66]	—	Arm I: 8.5 [95% CI, 6.6–11.9] Arm II: 10.1 [95% CI, 7.5–11.8] HR 1.25 [95% CI, 0.85–1.84]; *p* = 0.25
Buckner 2001 and Taylor 1998 [27, 28]	IFN‐alpha + BCNU	BCNU alone	Arm I: 300 days Arm II: 357 days *p* = 0.60	—	—
Harada 1996 [37]	IFN‐beta + MCNU	MCNU alone	—	One‐year survival rate: Arm I: 50.0% Arm II: 85.7% 2‐year survival rate: Arm I: 8.3% Arm II: 46.4% 5‐year survival rate: Arm I: 0.0% Arm II: 7.1%	—
Yoshida 1994 [18]	IFN‐beta + ACNU	ACNU alone	—	3‐year: both 30% 5‐year: both 18% *p* < 0.05	—

#### 3.4.2 Survival Rate

Guo et al. investigated the effect of interferon‐alpha on the median 2‐year and 5‐year OS rates. It was observed that this adjuvant therapy increased the median OS rates in the intervention group (adjuvant therapy plus TMZ) compared to the control group (TMZ alone).

Harada et al. investigated the effect of interferon‐beta adjuvant therapy compared to MCNU alone. It was demonstrated that adjuvant treatment had no impact on survival rates in glioma patients.

Yoshida et al. investigated the effect of interferon‐beta therapy (IFN + ACNU) and ACNU alone on 3‐year and 5‐year survival rates. It concluded that this adjuvant therapy had no significant impact on either the 3‐year or 5‐year survival rates (Table 4).

#### 3.4.3 Progression‐Free Survival (PFS)

Guo et al. investigated the effect of interferon therapy on median PFS, reporting no significant difference between the two arms of the study. In the WHO subgroup analysis, it was demonstrated that this adjuvant therapy had a significant effect on patients with grade 3 glioma. Still, no positive effects were observed in patients with grade 4 glioma. The median 2‐year and 5‐year PFS rates were better in the intervention group compared to the control.

Wakabayashi et al. demonstrated that IFN‐beta therapy had no significant effect on the median PFS of glioma patients.

Buckner and Taylor et al. studied the effect of IFN‐alpha adjuvant therapy on median PFS, reporting that IFN therapy did not have any significant effects compared to BCNU alone (*p* = 0.47) (Table 4).

#### 3.4.4 Time to Tumor Progression (TTP)

Buckner and Taylor et al. reported that the median TTP was shorter in the intervention group (IFN‐alpha + BCNU; 148 days) compared to the control group (BCNU alone; 169 days).

As shown in the Harada study, the median TTP of glioma patients decreased compared to the control group as the effect of interferon‐beta adjuvant therapy (IFN + MCNU: 6.9 ± 1.4 and MCNU alone: 8.5 ± 2.0 months) (Table 4).

#### 3.4.5 Adverse Events (Toxic Effects)

Guo et al. observed toxic effects, including hematological (anemia, leukopenia, and thrombocytopenia), flu‐like (fever, nausea, and fatigue/lethargy), epileptic, hepatic, and dermal toxicities as a result of interferon alpha‐immunotherapy.

The observed adverse events, as reported in the Wakabayashi study, included hematological (lymphopenia and neutropenia), flu‐like (fever, nausea, and vomiting/emesis), hepatic, and dermal toxicities. Epilepsy was not seen as the effect of interferon‐beta adjuvant therapy in the studied population.

All the toxic effects were seen, as the interferon adjuvant therapy, in the Buckner and Taylor study, including hematological (leukopenia and thrombocytopenia), flu‐like (fever, nausea, emesis, fatigue, myalgia, chill, pulmonary, and headache), epilepsy, and hepatic events. Any dermal toxicity was not seen in this study.

The observed side effects in the Harada study included hematological (leukopenia and thrombocytopenia), flu‐like (fever, nausea), and hepatic events.

#### 3.4.6 MGMT Subgroups

Guo (2023) and Natsume (2020) investigated the effect of interferon adjuvant therapy in MGMT (O6‐methylguanine‐DNA methyltransferase) subgroups on the treatment response of glioma patients, thereby studying variations in OS and PFS. Guo et al. reported that the median OS and median PFS were longer in all studied groups (as mentioned in Table 5). However, due to the P‐value, only the median OS of the unmethylated subgroup has valid results in the society. Natsume et al. concluded that the median OS of the unmethylated subgroup increased as a result of interferon therapy, but the median OS of the methylated subgroup remained unchanged (Table 5).

**TABLE 5:** MGMT subgroups.

Subgroups	MGMT unmethylated						MGMT methylated					
	OS			PFS			OS			PFS		
Study	IFN + TMZ	TMZ	HR	IFN + TMZ	TMZ	HR	IFN + TMZ	TMZ	HR	IFN + TMZ	TMZ	HR
Guo 2023 [19]	24.7 [95% CI, 20.5–28.8]	17.4 [95% CI, 14.1–20.7]	0.57 [95% CI, 0.37–0.87], *p* = 0.008	14.8 [95% CI, 11.6–18.0]	12.6 [95% CI, 11.8–13.5]	0.68 [95% CI, 0.46–1.02], *p* = 0.06	28.3 [95% CI, 17.4–39.2]	22.4 [95% CI, 19.2–25.6]	0.77 [95% CI, 0.49–1.21], *p* = 0.25	14.7 [95% CI, 8.7–20.7]	14.4 [95% CI, 11.9–16.9]	0.93 [95% CI, 0.60–1.43], *p* = 0.72
Natsume 2020 [36]	—	—	*p* = 0.40	—	—	—	—	—	*p* = 0.20	—	—	—

## 4 Data Analysis

### 4.1 OS and PFS

**FIGURE 4:**
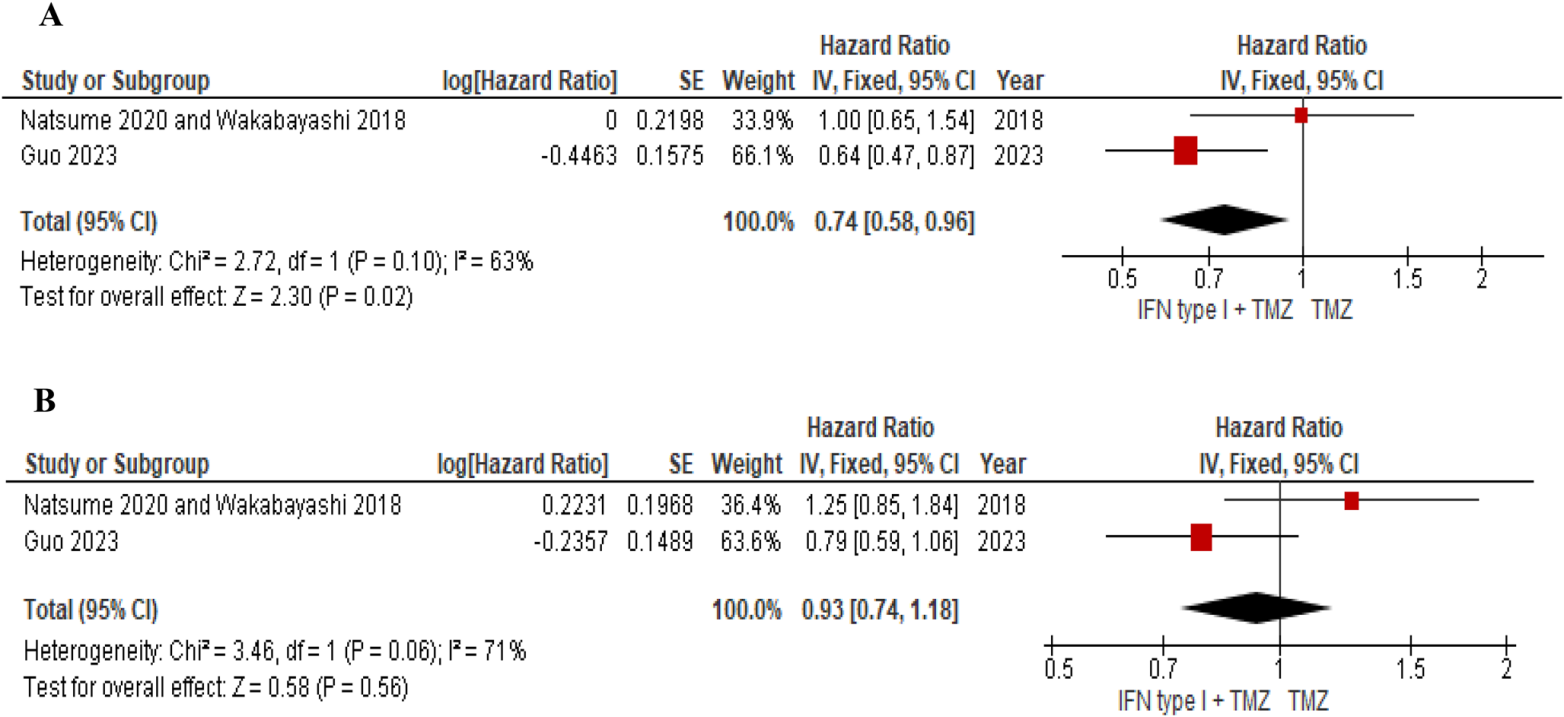
(A) The effect of interferon therapy on the median OS, (B) The effect of interferon therapy on the median PFS.

Both Guo 2023 and Natsume 2020, Wakabayashi 2018 studied the effect of interferon adjuvant therapy on OS and PFS. Guo et al. opposite to Natsume 2020, Wakabayashi 2018 reported that this therapy had a positive effect on the mean OS of glioma patients. Due to meta‐analysis, a significant effect of IFN therapy was obtained (HR = 0.74, 95% CI [0.58, 0.96]; *p* = 0.02). Based on *p* = 0.10 and $I^{2}$ = 63%, these studies exhibited substantial heterogeneity. This heterogeneity could originate from several sources. The most significant factor is variation in IFN subtypes, as mentioned, Guo et al. investigated IFN‐alpha, and Wakabayashi et al. investigated IFN‐beta. Additionally, different glioma subtypes, chemotherapy regimen, and mean age may also contribute to increased heterogeneity (Figure 4). The positive effect of the intervention on PFS was observed in the Guo study, but the Natsume and Wakabayashi study indicated the adverse effect of supplement therapy. Finally, data analysis showed a positive impact on PFS of glioma patients (HR = 0.93, 95% CI [0.74, 1.18]). Based on *p* = 0.56, the result is not statistically significant. Substantial heterogeneity (*I* ^2^ = 71%) was observed, likely due to variation in glioma subtypes, IFN variants, and chemotherapy regimens (Figure 4).

## 5 Adverse Events

**FIGURE 5:**
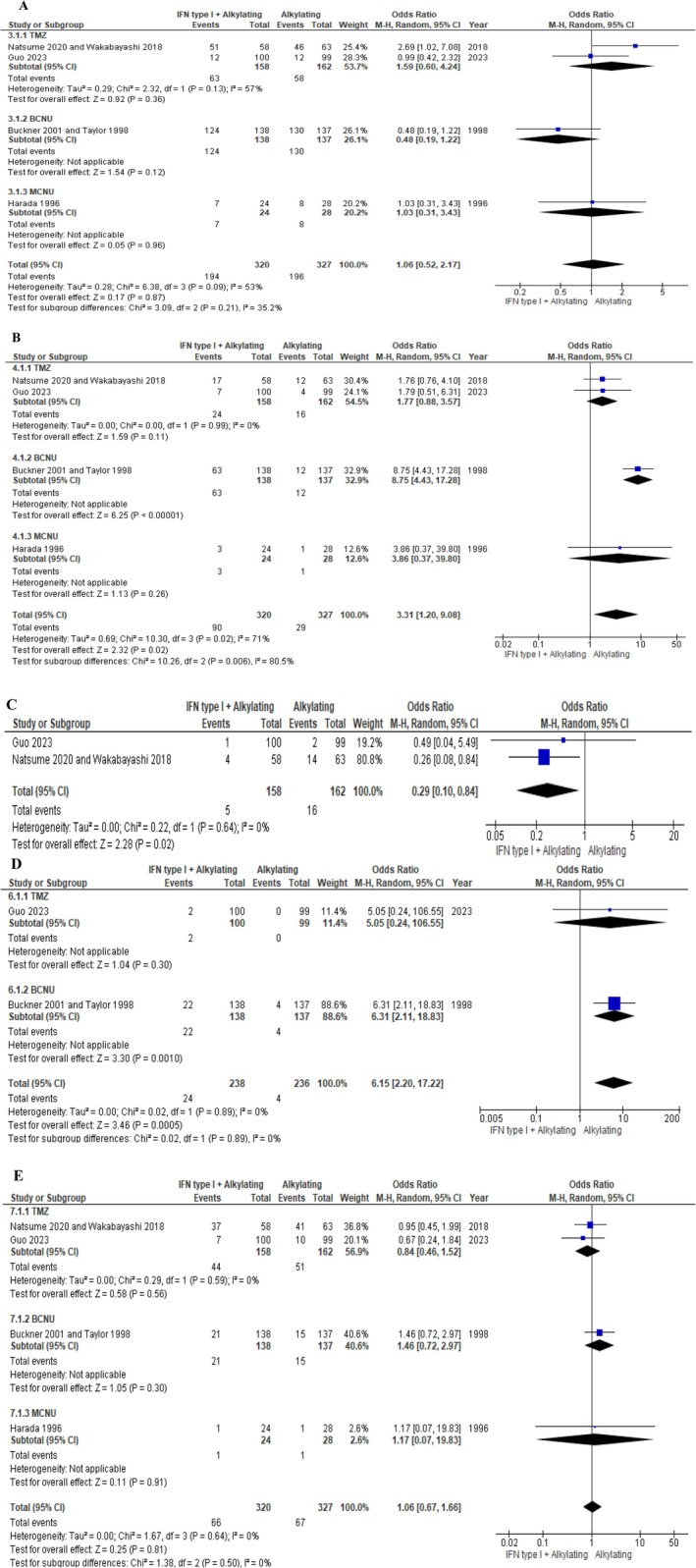
Adverse events: (A) Hematological (B) Flu‐like (C) Dermatological (D) Neurological (E) Hepatic.

Hematological toxicity was observed as the effect of interferon therapy in combination with three alkylating drugs (TMZ (Guo and Natsume, Wakabayashi), BCNU (Buckner, Taylor), and MCNU (Harada)). The worsening of this adverse event was observed in TMZ and MCNU (odds ratio = 1.59, *p* = 0.36, and odds ratio = 1.03, *p* = 0.96, respectively) subgroups, unlike the BCNU subgroup (odds ratio = 0.48, *p* = 0.12). Overall results demonstrated that this intervention did not have any hematological adverse effect on glioma patients (odds ratio = 1.06, 95% CI [0.52, 2.17]; *p* = 0.87). Due to *I* ^2^ = 53%, moderate heterogeneity was seen among the studies (Figure 5).

Flu‐like events were developed by the interaction of the interferon with alkylating drugs (TMZ (Guo and Natsume, Wakabayashi), BCNU (Buckner, Taylor), and MCNU (Harada)). All studies demonstrated that interferon therapy could cause Flu‐like adverse events in glioma patients. The ultimate result of the data analysis indicated that Flu‐like adverse events were effectively caused by the supplement therapy in glioma patients (odds ratio = 3.31, 95% CI [1.20, 9.08]; *p* = 0.02). In consideration of *I* ^2^ = 71%, the studies were substantially heterogeneous (Figure 5).

Analysis revealed that the hematological and flu‐like adverse events exhibited remarkable heterogeneity. This variation is likely due to differences in the types of interferon, chemotherapy regimens, and glioma subtypes.

Dermatological events were just investigated by Guo, Natsume, and Wakabayashi studies as adverse effects of interferon therapy with alkylating drug (TMZ). As a result of the meta‐analysis, a reduction in dermatological side effects of chemotherapy was indeed observed (odds ratio = 0.29, 95% CI [0.10, 0.84]; *p* = 0.02). The selected studies for data analysis were completely homogeneous (*I* ^2^ = 0%). [Figure 5].

Epilepsy was checked as a neurological adverse event of interferon adjuvant therapy in combination with alkylating drugs (TMZ (Guo), BCNU (Buckner, Taylor)). Neurological complications in glioma patients were reported in both subgroups. The overall result indicated that interferon therapy significantly increased the risk of epilepsy occurrence in glioma patients (odds ratio = 6.15, 95% CI [2.20, 17.22]; *p* = 0.0005). Complete homogeneity of the studies was observed with attention to *I* ^2^ = 0% (Figure 5).

Interferon adjuvant therapy with TMZ had a positive effect (odds ratio = 0.84) compared to adjuvant therapy with BCNU and MCNU (odds ratios = 1.46 and 1.17, respectively) on the hepatic events of glioma patients. In attention to meta‐analysis, the considerable hepatic toxic effect of this adjuvant therapy was not seen in chemotherapeutic glioma patients (odds ratio = 1.06, 95% CI [0.67, 1.66]; *p* = 0.81), homogeneity of selected studies was approved by considering *I* ^2^ = 0% (Figure 5).

## 6 Discussion

This study represents the first systematic review and meta‐analysis evaluating all randomized controlled trials investigating the effect of interferon type I in combination with alkylating drugs compared to alkylating drugs alone in newly diagnosed glioma patients.

The treatment process in all studies included radiation and chemotherapy, in which interferon type I was used as an adjuvant therapy with TMZ, ACNU, BCNU, and MCNU as alkylating drugs. Molecular and cellular studies suggested that interferon adjuvant therapy combined with standard chemotherapy increased tumor cell death and the expression level of the P53 encoding gene. Additionally, interferon downregulated the MGMT expression and improved the cell response to TMZ [38, 39]. Although all the mentioned compounds were alkylating drugs, they exhibited different effects on patients when combined with interferon type I. In this regard, interferon type I adjuvant therapy with TMZ resulted in synergistic effects on lifespan development. BCNU and MCNU had an antagonistic impact in combination with this supplemental therapy. ACNU with interferon adjuvant therapy was investigated only in Yoshida 1994, who concluded that this adjuvant therapy had a neutral effect on the lifespan of glioma patients.

Despite observations of the adjunct effect of type I interferons on GBM, a detailed review of the specific subtypes of type I interferons could be valuable. IFN type I can influence tumors by affecting both intrinsic and extrinsic factors. For example, high levels of PD‐L1 inhibit cytotoxic responses that enhance IFN‐ related resistance signature, which can prevent cancer cell death [40]. Shen et al. demonstrated that interferon type I can enhance glioma stem‐like cells (GSCs) by modulating MGMT expression, reducing resistance to temozolomide. Additionally, these interferons can suppress NF‐κB by inducing p53 pathways [41]. On the extrinsic pathways, IFN beta suppresses the growth of glioma stem cells by downregulating cell proliferation and ribosome pathways, in addition to reducing sphere formation and migratory signature by stimulating GSCs. Interferon beta also impacts GSCs by upregulating XAF1 and TRAIL death ligands [42, 43, 44]. Furthermore, interferon alpha can improve the activity of macrophages and natural killers, prevent blood vessel formation, and decrease tumor proliferation [19, 45].

As a result of the data analysis, it was shown that the median OS was significantly increased by interferon therapy while the PFS improvement did not reach statistical significance. These studies showed that the combination of interferon with alkylating drugs had a more synergistic effect on participants with lower‐grade gliomas and younger ages [19, 26]. Unmethylated MGMT is related to the resistance of gliomas to standard chemotherapy (temozolomide) and lower OS and PFS [36, 46]. Based on the reported data, interferon adjuvant therapy reduced the resistance to TMZ and increased the median OS in glioma patients [19, 36].

The discrepancies from Guo 2023 and Natsume 2020, and Wakabayashi 2018 on the OS and PFS may originate from several factors. First, differences may arise from IFN subtype; IFN alpha has a more positive effect with alkylating drugs compared to IFN beta. Second, as mentioned in Table 2, the median age of participants in Guo's study is lower than Wakabayashi's study. This suggests that the therapy may be more effective in younger patients. Third, as mentioned by Guo et al., the presence of residual disease can affect the outcomes. Wakabayashi's subgroup analysis also supports the idea that IFN beta may benefit patients without residual tumors. Fewer residual cases may have positive effects on the outcomes, indicating that the treatment is likely to be more effective in these patients.

Alkylating drugs, despite their lifespan efficacy, had some adverse effects, and interferon supplement therapy could have different effects (development or reduction) on them, or specific adverse events could be caused by this supplement therapy [47]. Adverse events include hematological, flu‐like, dermatological, neurological, and hepatic toxicities. In this way, interferon adjuvant therapy significantly increased the odds of flu‐like and neurological events, but it effectively reduced the occurrence probability of dermatological events, as analyzed. Notably, the hematological and hepatic toxic effects of interferon therapy were not approved by the analysis.

The mechanism of Flu‐like adverse events is linked to the release of cytokines such as tumor necrosis factor‐α, interleukin‐1, and interleukin‐6, which activate receptors in the hypothalamus [48]. Hematological adverse events stem from the inhibitory effect of interferons on growth, leading to myelosuppression [49]. Additionally, interferons induce the upregulation of adhesive molecules on leukocytes and endothelial cells, enhancing the adhesion of leukocytes to the endothelium and altering their redistribution in the body [50, 51]. Neurological and psychiatric effects are associated with pro‐inflammatory cytokines and somatic side effects that induce sickness behavior, a phenomenon better studied in animal models (the reaction of animals to infection or inflammation). The hepatic effects remain less understood, but most of them are associated with autoimmune hepatitis [52]. Other adverse events include renal adverse events and cardiovascular adverse events, which are associated with disrupted mitochondrial function and nerve inflammation. Endocrine events, such as de novo diabetes mellitus, are associated with autoimmunity responses [53, 54, 55, 56, 57].

According to the study, significant heterogeneity was observed in this context. Parameters such as the type of glioma, the location of tumors, and the ECOG performance of patients affected the surveillance outcomes. As mentioned, each type of interferon alpha and beta has a specific characteristic in addressing malignant glioma, making it beneficial for future studies to examine these interferons separately. Additionally, the different protocols used in the studies contributed to this heterogeneity. Furthermore, the introduction of new alkylating drugs over time has played a vital role in extending the lifespan of glioma patients, further contributing to the heterogeneity among studies. Additionally, due to environmental, genetic, and healthcare system differences, as well as the fact that most included studies were conducted in Asian populations, the generalizability of the findings to other ethnic or racial groups may be limited.

Therefore, finding demonstrates that type I interferons, particularly when combined with TMZ, especially among younger individuals and those with lower‐grade tumors, may improve glioma patients' OS. However, the effects of type I interferon subtypes vary: while interferon‐beta provides modest benefits due to its mechanistic advantages in targeting glioma stem cells and enhancing chemosensitivity, interferon‐alpha appears more helpful. Future studies should include RCTs that directly compare interferon‐alpha and interferon‐beta as adjuvants to novel alkylating agents like TMZ. These trials should stratify patients based on tumor grade, molecular markers (e.g., MGMT methylation, p53 status), tumor location, and performance status. Furthermore, studies should also be conducted in diverse geographical regions, including North America, Europe, and Africa, to determine whether the observed benefits of interferon therapy extend across racial and ethnic populations. Additionally, studies investigating the optimal route of interferon administration (systemic vs. intratumoral or gene therapy‐based) and its long‐term toxicity profiles will be crucial in defining its role in glioma therapy.

## 7 Conclusion

The systematic review and meta‐analysis demonstrate that type I interferons, when utilized as adjuvant therapy alongside alkylating agents (particularly temozolomide), could prolong the OS in newly diagnosed glioma patients, especially among younger individuals and those with lower‐grade tumors. However, the therapeutic benefits vary depending on the interferon subtypes and alkylating agents used. Due to the ability of interferon‐beta to inhibit the glioma cell cycle and enhance chemosensitivity, greater therapeutic advantages have been observed. However, Interferon‐alpha demonstrated limited efficacy alone; its combination with alkylating drugs provided survival benefits. Adverse effects, including flu‐like and neurological symptoms, were more common with interferon therapy; however, it decreased dermatological toxicities.

Given the studies' heterogeneity (interferon subtypes and delivery routes) and limited race diversity, further RCTs are required to clarify the role of type I interferons in modern glioma treatment protocols.

## Author Contributions

**Nima Goudarzi:** conceptualization, investigation, writing – original draft, methodology, validation, visualization, writing – review and editing, formal analysis. **Abolfazl Sam Daliri:** conceptualization, investigation, writing – original draft, methodology, validation, visualization, writing – review and editing, formal analysis. **Arshia Harati:** writing – original draft, methodology, validation, visualization, writing – review and editing, formal analysis. **Shabnam Soheil Nader:** formal analysis, investigation, funding acquisition, writing – original draft, conceptualization, visualization, validation, methodology, writing – review and editing, data curation, supervision, resources, project administration. **Kourosh Kabir:** data curation, supervision, resources, investigation, funding acquisition, writing – original draft, methodology, validation, visualization, writing – review and editing, formal analysis, project administration.

## Funding

The authors have nothing to report.

## Ethics Statement

The authors have nothing to report.

## Consent

The authors have nothing to report.

## Conflicts of Interest

The authors declare no conflicts of interest.

## Data Availability

The data that support the findings of this study are available from the corresponding author upon reasonable request.
